# Trauma, attachment style, and somatization: a study of women with dyspareunia and women survivors of sexual abuse

**DOI:** 10.1186/s12905-018-0523-2

**Published:** 2018-01-30

**Authors:** Michal Granot, Yoram Yovell, Eli Somer, Ahuva Beny, Ronit Sadger, Ronit Uliel-Mirkin, Yaara Zisman-Ilani

**Affiliations:** 1 Faculty of Social Welfare & Health Sciences, University of Haifa, Mount Carmel, 31905 Haifa, Israel; 2 Institute for the Study of Affective Neuroscience, University of Haifa, Haifa, Israel; 3 School of Social Work, Faculty of Social Welfare and Health Sciences, University of Haifa, Haifa, Israel; 4 Multidisciplinary Treatment Center for Victims of Sexual Abuse and Head of the Psychiatric Unit, Bnaiy-Zion Medical Center, Haifa, Israel; 5 Multidisciplinary Treatment Center for Victims of Sexual Abuse, Bnaiy-Zion Medical Center, Haifa, Israel; 6 The Center for Mental Health Research, Practice and Policy, University of Haifa, Haifa, Israel; 7 Department of Rehabilitation Sciences, College of Public Health, Temple University, Philadelphia, PA USA

**Keywords:** Sexual abuse, Attachment, Pain, Somatic psychology, Trauma

## Abstract

**Background:**

Evidence points toward shared characteristics between female survivors of sexual abuse and women with dyspareunia. This study explored, for the first time, similarities and differences between women who were exposed to sexual abuse to those with dyspareunia, in order to examine whether insecure attachment styles and high somatization level are associated with trauma among women with dyspareunia.

**Methods:**

Attachment styles were explored using the Experience in Close Relationships Scale to reflect participants’ levels of anxiety and avoidance. Somatization was assessed using the Brief Symptom Inventory focusing on the frequency of painful and non-painful bodily complaints. Trauma was categorized into three levels: sexual trauma, nonsexual trauma, and no trauma.

**Results:**

Sexually abused (SA) women (*n* = 21) compared to women with dyspareunia (dys) (*n* = 44) exhibited insecure attachment styles, as expressed by high levels of avoidance (SA 4.10 ± 0.99 vs. dys 3.08 ± 1.04, *t*_(61)_ = 2.66, *p* = .01) and anxiety (SA 4.29 ± 1.22 vs. dys 3.49 ± 1.04, *t*_(61)_ = 3.61, *p* = .001), and higher somatization (21.00 ± 8.25 vs. 13.07 ± 7.57, *t*_(59)_ = 3.63, p = .001). Attachment and somatization level did not differ significantly between women with dyspareunia without trauma to those with nonsexual trauma.

**Conclusions:**

Our findings emphasized the unique role of sexual trauma as a contributing factor to the augmentation of perceived bodily symptoms and to insecure attachment style. This illuminates the importance of disclosing previous sexual abuse history among women with dyspareunia.

## Background

Sexual Abuse (SA) was found to account for the majority of traumatic events among women [1, 2]. Exposure to traumatic events in general, and to SA in particular, has been shown to produce many negative short- and long-term consequences, including physical and mental health problems, such as alexithymia and emotional dysregulation [3–8] Exposure to sexual or physical abuse during childhood is associated with an increased risk of chronic pain in adulthood [9, 10]. Even stressful events which are not often considered traumatic have the potential for long-term alteration of pain modulation [11].

Studies have indicated that women who were exposed to SA are often characterized by a higher incidence of physical complaints, pain disorders, vaginismus, and higher prevalence of sexual dysfunction [5]. This includes elevated levels of sexual distress, pelvic floor complaints and dyspareunia, defined as pain associated with intercourse [12–17]. Interestingly, several studies reported that women with dyspareunia present similar characteristics as women with a history of SA, including vaginismus [18, 19], difficulties in sexual functioning and intimate relationships [20], and greater severity of sexual pain [21]. Previous studies in the field of pelvic pain disorder have tended to rely upon a group of healthy women as the control without actively comparing characteristics of women survivors of SA and those with dyspareunia, in order to better understand inter-group similarities and differences. This comparison is critical, as both groups of women may demonstrate insecure attachment styles, while the role of trauma of any kind in dyspareunia has yet to be fully elucidated.

It is widely accepted that patterns of adult intimate relationships are shaped by individual attachment styles [22, 23]. Studies on female sexuality suggested that anxious and avoidant attachment styles are associated with less satisfying sexual relationships, higher levels of sexual dysfunction [12, 24], less sexual arousal [25], problems with lubrication [26], lack of orgasm [27], and pain associated with intercourse [28]. In addition, trauma during childhood was found to influence levels of somatization by fostering insecure attachment in adulthood [29]. Previous work has also shown that women who suffered from dyspareunia were found to be characterized by less secure attachment styles and greater levels of somatization [30–33]. In the same vein, a less secure attachment style has been reported to be associated with elevated pain sensitivity among patients suffering from chronic pain [34].

The possibility that attachment styles and somatization may be associated with past trauma, which in turn may be linked with present-day pain during intercourse, may widen the scope of understanding regarding the underlying processes in the development of chronic pelvic pain disorders, with the hope for a resultant more comprehensive clinical approach. The present study aimed to explore the possibility that past sexual and nonsexual trauma among women is associated with their attachment style and their level of somatization. More specifically, we aimed to investigate whether dyspareunic women who were exposed to nonsexual trauma demonstrate a different manifestation of somatic symptoms and attachment style as compared with female survivors of sexual abuse.

## Methods

### Participants and procedure

The study sample was consisted of 65 women, comprised of two groups: survivors of SA and women with dyspareunia. The first group, “women who experienced sexual abuse (SA)”, consisted of 21 women who were recruited from a treatment center for survivors of sexual abuse as part of a larger study. These women had been exposed to severe sexual trauma, mainly during childhood, as well as to other nonsexual traumatic events, and had participated in a therapy program tailored to SA for at least three months prior to data collection (for more details: [35]). The second group included 45 women who reported painful intercourse, i.e. dyspareunia, who participated in a previous study that compared women with dyspareunia with healthy controls (for more details: [33]). This group was recruited via community gynecological clinics and reported no history of systemic disease, endometriosis or any hormonal disorders. After recruitment, each of the women in this group was asked whether she had been exposed to a traumatic event of any kind, such as being involved in a car accident, natural disaster, war, or sexual abuse. We have used the first part of the ‘Posttraumatic Stress Diagnostic Scale’ [36] which assesses various potential conditions that may be perceived as traumatic events in order to obtain the information about past exposure to trauma. Of the 45 women, 18 reported past trauma (nonsexual), and 26 had no previous experience of trauma. For the purpose of the current study, we excluded from the original sample those who reported sexual trauma (*N* = 1), leaving a total sample size of 44 in this group.

### Assessment tools

All participants were asked to fill in two questionnaires: the first assessed attachment style, and the second measured level of somatization. Since the somatization questionnaire does not relate specifically to a chronic pain disorder, we added a question about, Dysmenorrhea, a common idiopathic pain disorder among women with dyspareunia, to better explore symptoms associated with pain. Thus, each woman reported her level of pain experienced during the first two days of their menstruation for the preceding 6 months.

#### Attachment styles

The Experience in Close Relationships Scale [37, 38] was used to assess attachment style, with 36 items measuring attachment anxiety and avoidance styles. Women rated the extent to which each item was descriptive of their experiences in close relationships on a 7-point Likert scale ranging from 1 (*not at all*) to 7 (*very much*). Eighteen items refer to attachment anxiety (e.g., “I worry about being abandoned”) and 18 items measure attachment avoidance (e.g., “I find it difficult to allow myself to depend on close relationship partners”). The reliability and validity of the scales have been repeatedly demonstrated in both English and Hebrew [33, 37, 38].

#### Level of somatization

The level of somatization was assessed using the short version of the Brief Symptom Inventory [39], which represents one factor in the Symptom Check List [40]. The questionnaire rates the frequency of complaints or symptoms in different areas of the body, including chest pain, headache, low back pain, vomiting, dizziness, flushes, or numbness. This multidimensional instrument is a 13-item self-report questionnaire on psychological distress and multiple aspects of psychopathology. It is often included in the evaluation of pain patients [41] and has been found appropriate for the Israeli population [33, 35].

### Statistical analyses

The statistical analyses were performed using IBM SPSS (version 21). Socio-demographic data, attachment styles, and level of somatization were compared between the SA and dyspareunia groups using independent sample *t*-tests. The dyspareunia group was then subdivided into two sub-groups; those with past trauma (nonsexual), and those without. ANOVA was used to determine whether the types of trauma (SA, nonsexual and no trauma) were associated with attachment styles and somatization by comparing the SA group with each of the two subtypes of dyspareunia. The level of significance was set at *p* < .05.

## Results

### Characteristics of the study sample

Overall no significant differences were observed between the two groups in most socio-demographic variables including level of education or employment status. For example, regarding years of education it was found that the dyspareunia group had a mean of 14.81 (SD = 1.90) and the SA group a mean of 13.90 (SD = 2.63; *t*_(29.22)_ = − 1.38, *p* > .05). However, it should be noted that the mean age of the SA group (M = 34.43, SD = 14.73) was higher as compared to the dyspareunia group (M = 25.48, SD = 3.27; *t*_(20.95)_ = 2.75, *p* < .05). This seems not to have a major clinical significance as the median age in both groups was the same (M_d_ = 26 years old). The mean duration of sexual abuse was 4.6 years (SD = 6.42). The mean age of reported sexual abuse onset was 9.87 years (SD = 6.7).

### Comparison between the SA and the dyspareunia groups

#### Attachment style

Two separate *t*-tests were performed to explore both dimensions of attachment style; level of anxiety and level of avoidance, between the SA group and the dyspareunia group. Results revealed a significantly less secure attachment style among the SA group, expressed by both higher levels of anxiety (M = 4.29, SD = 1.22 for the SA group; M = 3.49, SD = 1.04 for the dyspareunia group), and avoidance [(M = 4.10, SD = 0.99 for the SA group, M = 3.08, SD = 1.04 for the dyspareunia group); (*t*_(61)_ = 2.66, *p* = .01 for the anxiety dimension), and (*t*_(61)_ = 3.61, *p* = .001 for avoidance dimension)].

#### Somatization

Women in the SA group demonstrated a higher level of somatization (M = 21.00, SD = 8.25) as compared with women in the dyspareunia group (M = 13.07, SD = 7.57; *t*_(59)_ = 3.63, p = .001). In addition, no significant differences (*p* > .05) were found between the two groups, nor between the three sub-groups, in relation to the prevalence of dysmenorrhea.

#### The role of trauma type in attachment dimensions and somatization

**Fig. 1:**
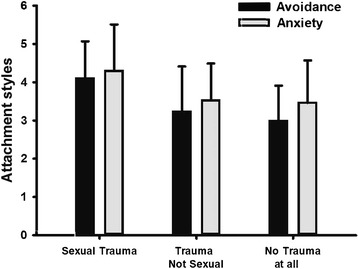
Differences in levels of avoidance and anxiety between three groups of women: with SA, with dyspareunia and a history of nonsexual trauma and with dyspareunia but without any history of trauma

In order to examine whether exposure to nonsexual trauma affects attachment styles and somatization, women exposed to SA were compared with two sub-groups of women with dyspareunia; those with a history of nonsexual trauma and those without any history of trauma. ANOVA analyses comparing the three groups for their attachment styles and level of somatization revealed significant differences between the three groups: f_(2,62)_ = 3.51, *p* = .036 for the anxiety dimension of attachment; f_(2,62)_ = 6.78, *p* = .002 for avoidance dimension of attachment; and f_(2,60)_ = 5.53, *p* = .001 for level of somatization. Post-hoc analyses using Fisher’s Least Significant Difference (LSD) revealed significant differences between the SA group and the two sub-groups of dyspareunia for anxiety, avoidance, and somatization (see Fig. 1). No significant difference was found between the two dyspareunia sub-groups.

## Discussion

This is the first study to compare women who had experienced SA with women with dyspareunia in order to examine their similarities and differences in regard to attachment styles and somatization. The study also investigated whether exposure to sexual vs. nonsexual trauma changes the psychological reaction expressed by tendency for higher anxiety and avoidance levels and somatic complaints among these women.

The main finding of the present study was that insecure attachment style, expressed by higher levels of anxiety and avoidance, as well as a high level of somatization were more prevalent among women survivors of SA compared to women with dyspareunia. These findings indicate that insecure attachment style and somatization were not affected by previous exposure to nonsexual trauma.

Consistent with previous publications that found a higher incidence of medically unexplained physical symptoms with no clear organic origin among individuals with an insecure attachment style [29, 42–44], our findings provide additional support that exposure to sexual trauma can impact both intimacy and negative experiences of the body due to pain or other physical symptoms. It should also be noted that dyspareunia and vaginismus are two sexual dysfunction conditions that are often associated, according to DSM IV criteria [5]. This concept has previously been demonstrated, arguing that sexual abuse in childhood should be treated as a multifaceted type of trauma, potentially leading to a comorbidity of PTSD and dissociative disorders [4]. It has also been suggested that somatization can be referred to as a Somatoform Dissociation, denoting phenomena that are manifestations of a lack of integration of somatoform experiences, reactions, and functions [16, 45]. We assume that among SA women, who suffer a great deal of emotional problems due to the exposure to sexual trauma, the high level of somatic symptoms may serve as an additional way to express their difficulties, as well as allowing them to communicate about their concerns. Another explanation may be attributed to the therapeutic process that the SA women underwent in the rehabilitation center for victims of sexual violence. As part of the therapy and treatment in this center, women were guided to acknowledge their bodily sensations as a key factor in their rehabilitation process. Thus, the higher self-report levels of somatization may serve as an additional path to express their distress as part of their treatment at the center.

In order to better address the existence of pelvic pain symptoms from a different perspective, women were also asked to report whether they suffer from dysmenorrhea as an additional indicator for a somatic symptom that is well known to characterize idiopathic pain disorder. Our findings show that the existence of dysmenorrhea, which represents different facet of somatic complaints, did not differ between groups. Future studies should closely assess the role of dysmenorrhea in the clinical manifestation of somatization among women who were victims of sexual abuse. It has been suggested that an insecure attachment style mediates the link between past trauma and current somatization level [29], to protect themselves from further harm by distancing themselves from intimate situations which may elicit uncomfortable thoughts and emotions. Therefore, attachment styles likely play a role in the manifestation of somatic complains following traumatic events. According to our findings, such an association is more relevant to survivors of SA who manifested insecure attachment styles and higher levels of somatization as compared to women with dyspareunia. Nevertheless, it should be noted that even impersonal traumatic events, such as natural disasters and accidents, or interpersonal trauma, such as assaults nave a negative effect during adulthood [46]. Furthermore, even exposure to indirect trauma in childhood, might cause conflict and feelings of guilt that may be expressed later on by higher pain symptoms reports [47].

This pilot study has several limitations to consider. First, it was exploratory and retrospective by nature, and this might have affected the ability to precisely recall the traumatic events that part of the women was exposed to. Since, the SA group was exposed to trauma at a young age (yet we did not collect additional information regarding the presence of other traumatic experiences in childhood); the ability to attain reliable reports should not be ignored. The SA women were recruited in a specific rehabilitation center for victims of sexual violence. Thus, we cannot ignore the possibility that the treatment and program at the center may also have affected their reports regarding the research variables. Second, the absence of the use of a standardized measure for female sexual functioning, such as the Female Sexual Function Index, reduced our ability to address the multidimensional nature of female sexual function and quality of life, thus limited the possibility to reveal additional dimensions of their sexual status. Third, our relatively small sample size restricted the ability to generalize our findings and to conduct additional statistical analyses. Future recruitment of a larger sample will allow the exploration of whether attachment style mediates between traumatic events and somatization. Third, we were unable to explore the study questions among men, although the prevalence of childhood SA among men is similar to that among women. Future research should explore the relationship between our research variables among men. Finally, we had not inquired about dyspareunia among participants in the SA group. Including an additional group of women who suffered from dyspareunia and previous sexual trauma will allow for a better understanding of the effect of trauma type among women suffering from dyspareunia.

## Conclusions

In conclusion, this study draws attention to the characteristics of attachment style and somatization among women survivors of SA and women with dyspareunia and emphasizes the unique impact of sexual trauma on current symptom severity versus nonsexual trauma and no trauma at all.

## Abbreviation

SA: Sexual Abuse

## References

[1] de Haas S, van Berlo W, Bakker F, Vanwesenbeeck I (2012). Prevalence and characteristics of sexual violence in the Netherlands, the risk of Revictimization and pregnancy: results from a National Population Survey. Violence Vict.

[2] Perkonigg A, Kessler RC, Storz S, Wittchen H-U (2000). Traumatic events and post-traumatic stress disorder in the community: prevalence, risk factors and comorbidity. Acta Psychiatr Scand.

[3] Cummings M, Berkowitz SJ, Scribano PV (2012). Treatment of childhood sexual abuse: an updated review. Current Psychiatry Reports.

[4] Lev-Wiesel R (2008). Child sexual abuse: a critical review of intervention and treatment modalities. Child Youth Serv Rev.

[5] Ciocca G, Limoncin E, Di Tommaso S, Gravina GL, Di Sante S, Carosa E (2013). Alexithymia and vaginismus: a preliminary correlation perspective. Int J Impot Res.

[6] Ciocca G, Limoncin E, Di TS, Mollaioli D, Gravina GL, Marcozzi A (2014). Attachment styles and sexual dysfunctions: a case-control study of female and male sexuality. Int J Impot Res.

[7] Cashmore J, Shackel R (2013). The long-term effects of child sexual abuse.

[8] Nerum H, Halvorsen L, Straume B, Sørlie T, Øian P (2013). Different labour outcomes in primiparous women that have been subjected to childhood sexual abuse or rape in adulthood: a case-control study in a clinical cohort. BJOG An Int J Obstet Gynaecol.

[9] Davis DA, Luecken LJ, Zautra AJ (2005). Are reports of childhood abuse related to the experience of chronic pain in adulthood? A meta-analytic review of the literature. Clin J Pain.

[10] Lampe A, Doering S, Rumpold G, Sölder E, Krismer M, Kantner-Rumplmair W (2003). Chronic pain syndromes and their relation to childhood abuse and stressful life events. J Psychosom Res.

[11] Tesarz J, Eich W, Treede R-D, Gerhardt A (2016). Altered pressure pain thresholds and increased wind-up in adult patients with chronic back pain with a history of childhood maltreatment. Pain.

[12] Stefanou C, McCabe MP. Adult attachment and sexual functioning: a review of past research: Review Adult Attachment and Sexual Functioning J Sex Med 2012;9:2499–2507. doi:10.1111/j.1743-6109.2012.02843.x. 10.1111/j.1743-6109.2012.02843.x 22759319

[13] Leclerc B, Bergeron S, Binik YM, Khalife S (2010). History of sexual and physical abuse in women with Dyspareunia: association with pain, psychosocial adjustment, and sexual functioning. J Sex Med.

[14] Desrochers G, Bergeron S, Landry T, Jodoin M (2008). Do psychosexual factors play a role in the etiology of provoked vestibulodynia? A critical review. J Sex Marital Ther..

[15] Dennerstein L, Guthrie JR, Alford S (2004). Childhood abuse and its association with mid-aged women’s sexual functioning. J Sex Marital Ther.

[16] Nijenhuis ERS (2000). Somatoform dissociation: major symptoms of dissociative disorders. J Trauma Dissociation.

[17] Masheb RM, Nash JM, Brondolo E, Kerns RD (2000). Vulvodynia: an introduction and critical review of a chronic pain condition. Pain.

[18] Postma R, Bicanic I, van der Vaart H, Laan E (2013). Pelvic floor muscle problems mediate sexual problems in young adult rape victims. J Sex Med.

[19] Reed BD. Dyspareunia and sexual/physical abuse. Female Sexual Pain Disorders. 2009:213–7. 10.1002/9781444308136.ch32.

[20] Bancroft J, Loftus J, Long JS (2003). Distress about sex: a National Survey of women in heterosexual relationships. Arch Sex Behav.

[21] Randolph ME, Reddy DM (2006). Sexual functioning in women with chronic pelvic pain: the impact of depression, support, and abuse. J Sex Res.

[22] Bowlby J (1988). A secure base : clinical applications of attachment theory.

[23] Hazan C, Shaver P (1987). Romantic love conceptualized as an attachment process. J Pers Soc Psychol.

[24] Mikulincer M, Goodman GS (Eds.). Dynamics of romantic love: Attachment, caregiving, and sex. Guilford Press. 2006. https://www.google.com/books?hl=en&lr=&id=eREk6ZyDC9YC&oi=fnd&pg=PA3&dq=dynamics+of+romantic+love&ots=3XwJx4NzAS&sig=Iy4LmJi1d58n6YaIATDT8cTgHK8. Accessed 10 Sep 2017.

[25] Birnbaum GE (2007). Beyond the borders of reality: attachment orientations and sexual fantasies. Pers Relatsh.

[26] Brassard A, Lussier Y, Shaver PR (2009). Attachment, perceived conflict, and couple satisfaction: test of a mediational dyadic model. Fam Relat.

[27] Cohen DL, Belsky J (2008). Individual differences in female mate preferences as a function of attachment and hypothetical ecological conditions. J Evol Psychol.

[28] Stephenson KR, Meston CM (2010). Differentiating components of sexual well-being in women: are sexual satisfaction and sexual distress independent constructs?. J Sex Med.

[29] Waldinger RJ, Schulz MS, Barsky AJ, Ahern DK (2006). Mapping the road from childhood trauma to adult Somatization: the role of attachment. Psychosom Med.

[30] Lackner JM, Gudleski GD, Blanchard EB (2004). Beyond abuse: the association among parenting style, abdominal pain, and somatization in IBS patients. Behav Res Ther.

[31] Landa A, Bossis AP, Boylan LS, Wong PS. Beyond the unexplainable pain: relational world of patients with somatization syndromes. J Nerv Ment Dis. 2012;200(5):413–22. http://journals.lww.com/jonmd/Abstract/2012/05000/Beyond_the_Unexplainable_Pain__Relational_World_of.8.aspx. Accessed 29 Jan 2018. 10.1097/NMD.0b013e3182532326 22551795

[32] Van Lankveld JJDM, Granot M, Weijmar Schultz WCM, Binik YM, Wesselmann U, Pukall CF, et al. Women’s sexual pain disorders. J Sex Med. 2010;7 1 PART 2:615–31. doi:10.1111/j.1743-6109.2009.01631.x. 10.1111/j.1743-6109.2009.01631.x 20092455

[33] Granot M, Zisman-Ilani Y, Ram E, Goldstick O, Yovell Y (2011). Characteristics of attachment style in women with dyspareunia. J Sex Marital Ther..

[34] Andrews NE, Strong J, Meredith PJ. Activity pacing, avoidance, endurance, and associations with patient functioning in chronic pain: a systematic review and meta-analysis. Arch Phys Med Rehabil. 2012;93 10.1016/j.apmr.2012.05.029. 10.1016/j.apmr.2012.05.029 22728699

[35] Granot M, Somer E, Zisman-Ilani Y, Beny A, Sadger R, Mirkin R, et al. Characteristics of response to experimental pain in sexually abused women. Clin J Pain. 2011;27:616–22. doi:10.1097/AJP.0b013e3182132963 [doi]. 10.1097/AJP.0b013e3182132963 21430522

[36] Foa EB. Posttraumatic stress diagnostic scale manual. Minneapolis, MN: National Computer Systems Pearson. Inc CIT0024. 1995.

[37] Brennan KA, Clark CL, Shaver PR. Self-report measurement of adult attachment: An integrative overview. In J. A. Simpson & W. S. Rholes (Eds.), Attachment theory and close relationships. New York: Guilford Press. 1998. pp. 46–76. http://psycnet.apa.org/psycinfo/1997-36873-002. Accessed 29 Jan 2018.

[38] Mikulincer M, Florian V (2000). Exploring individual differences in reactions to mortality salience: does attachment style regulate terror management mechanisms?. J Pers Soc Psychol.

[39] Derogatis LR, Melisaratos N (1983). The brief symptom inventory: an introductory report. Psychol Med.

[40] Derogatis LR, Cleary PA (1977). Confirmation of the dimensional structure of the scl-90: a study in construct validation. J Clin Psychol.

[41] Hardt J, Gerbershagen HU, Franke P (2000). The symptom check-list, SCL-90-R: its use and characteristics in chronic pain patients. Eur J Pain.

[42] Ciechanowski P, Russo J, Katon W, Von Korff M, Ludman E, Lin E (2004). Influence of patient attachment style on self-care and outcomes in diabetes. Psychosom Med.

[43] Noyes Jr., RN, Stuart SP, Langbehn DR, Happel RL, Longley SL, Muller BA, Yagla SJ. Test of an interpersonal model of hypochondriasis. Psychosom Med. 2003;65(2):292–300. http://journals.lww.com/psychosomaticmedicine/Abstract/2003/03000/Test_of_an_Interpersonal_Model_of_Hypochondriasis.16.aspx. Accessed 29 Jan 2018. 10.1097/01.psy.0000058377.50240.64 12651997

[44] Taylor RE, Mann AH, White NJ, Goldberg DP (2000). Attachment style in patients with unexplained physical complaints. Psychol Med.

[45] Nijenhuis ER, van Dyck R, ter Kuile MM, Mourits MJ, Spinhoven P, van der Hart O (2003). Evidence for associations among somatoform dissociation, psychological dissociation and reported trauma in patients with chronic pelvic pain. J Psychosom Obstet Gynaecol.

[46] Allen J. Traumatic Relationships and Serious Mental Disorders 2003. doi:10.4088/JCP.v63n0915f.

[47] Unternährer I, Minder CE, Adler RH. Gender and the relationship between traumatic childhood experiences and pain in adulthood. Swiss Med Wkly. 2006;136:637–642. doi:2006/39/smw-11357. 10.4414/smw.2006.11357 17086510

